# Uric acid levels correlate with disease activity in growth hormone-secreting pituitary adenoma patients

**DOI:** 10.3389/fendo.2023.1230852

**Published:** 2023-09-25

**Authors:** Caiyan Mo, Han Chen, Fang Wang, Ying Guo, Yao Wang, Tao Tong, Liyong Zhong

**Affiliations:** Department of Endocrinology, Beijing Tiantan Hospital, Capital Medical University, Beijing, China

**Keywords:** uric acid, growth hormone-secreting pituitary adenoma, growth hormone, insulin-like growth factor-1, disease activity

## Abstract

**Objective:**

Few studies reported the effects of growth hormone-secreting pituitary adenoma (GHPA) on uric acid (UA) metabolism and the relationship between growth hormone (GH)/insulin-like growth factor-1 (IGF-1) levels and UA are controversial. This study aimed to evaluate the relationship between IGF-1 and UA in patients with GHPA and to further clarify whether UA levels are associated with GHPA disease activity by follow-up.

**Methods:**

A longitudinal study of 424 GHPA patients presenting to Beijing Tiantan Hospital, Capital Medical University between January 2015 and January 2023 was conducted. Spearman’s correlation tests were performed to examine the relationship between IGF-1 and UA at baseline. Univariate and multivariate linear regression analysis was conducted to investigate the independent association between UA and IGF-1. Changes in postoperative IGF-1 and UA levels were followed prospectively, and the differences in UA levels between the biochemical remission and nonremission groups were compared.

**Results:**

At baseline, male patients, the lower the age, the higher the IGF-1 and body mass index (BMI), and the higher the UA levels. IGF-1 was significantly associated with UA after controlling for sex, age, and BMI (r = 0.122, *P* = 0.012). In adjusted multiple linear regression analysis, IGF-1 was independently associated with UA, and UA levels increased significantly with increasing IGF-1. During postoperative follow-up, UA decreased gradually as IGF-1 levels decreased. At 12 months postoperatively, UA levels were significantly lower in the biochemical remission group than in the nonremission group (*P* = 0.038).

**Conclusions:**

In patients with GHPA, UA levels are associated with disease activity. Changes in UA levels should be taken into account in the comprehensive treatment of GHPA, patients presenting with HUA should be given lifestyle guidance and appropriate urate-lowering treatment according to their condition to better improve their prognosis.

## Introduction

Growth hormone-secreting pituitary adenoma (GHPA) is a rare disease that results from the uncontrolled secretion of growth hormone (GH) by a functional pituitary adenoma, leading to a series of characteristic clinical manifestations. The incidence rate of GHPA has been reported to be 0.38 [95% confidence interval (CI): 0.32-0.44] cases per 100 000 person-years, with a pooled prevalence of 5.9 (95% CI: 4.4-7.9) per 100 000 persons (1). Excessive GH stimulates the liver to produce insulin-like growth factor-1 (IGF-1) (2), and long-term overproduction of GH and IGF-1 promotes excessive hyperplasia of soft tissues, bone, and cartilage throughout the body, leading to the typical signs and symptoms of gigantism or acromegaly in patients, and can cause multi-organ/system complications such as cardiovascular, respiratory, and digestive systems (3, 4). Besides, GHPA also leads to metabolic abnormalities in the body, and the most studied effects are on glucose metabolism and lipid metabolism (5–7), however, the effects on uric acid (UA) metabolism have been poorly studied. Moreover, the relationship between GH/IGF-1 levels and UA in different pathophysiological states is controversial. In healthy individuals, acute infusion of IGF-1 decreased UA (8). Similarly, in nondiabetic adult subjects, IGF-1 and UA were inversely correlated (9). In obese children and adolescents, reduced peak stimulated GH was associated with hyperuricemia (HUA) (10). However, in children with idiopathic short stature (ISS) treated with recombinant human growth hormone (rhGH) replacement, UA concentrations showed a non-linear relationship with IGF-1 standard deviation score (IGF-1 SDS) (11). Besides, studies have reported no significant effect of rhGH administration on UA levels in boys with obesity and nonalcoholic fatty liver disease (NAFLD) (12).

Pulsatile GH secretion by anterior pituitary somatotroph cells is normally under dual control exerted by hypothalamic peptides, including stimulation by growth hormone-releasing hormone and inhibition by somatostatin. In healthy individuals, GH is secreted episodically, predominantly during slow-wave sleep or during exercise (13). Moreover, GH is a stress hormone, so basal serum GH values do not reflect the functional status of the GH/IGF-1 axis, whereas IGF-1 levels are relatively stable in circulation and can be used as an important indicator to assess disease activity (14, 15). Therefore, this study was aimed at analyzing the association between serum UA levels and IGF-1 and the factors influencing UA levels in patients with GHPA, and to further clarify whether UA levels in patients with GHPA are related to disease activity by measuring changes in postoperative serum UA levels relative to the decrease in IGF-1 levels.

## Methods

### Study design and participants

We recruited a total of 483 patients with GHPA who presented to Beijing Tiantan Hospital, Capital Medical University between January 2015 and January 2023. The inclusion criteria were as follows: (1) meeting the diagnostic criteria for GHPA, including clinical features of gigantism or acromegaly; unsuppressed serum GH to less than 1 ng/mL after oral glucose tolerance test of 75 g glucose; elevated serum IGF-1 levels after sex- and age-adjusted; and pituitary adenomas confirmed by contrast-enhanced magnetic resonance imaging. (2) sufficient available medical records. The exclusion criteria were as follows: (1) pituitary adenomas that co-secret other hormones (prolactin, thyroid stimulating hormone) (n = 32). (2) patients who combined malignancy, severe hepatic or renal insufficiency (n = 9). (3) patients who taking drugs that affect UA levels (n = 18). A total of 424 patients with GHPA were eventually included. Basic information of each patient was recorded, including age, sex, height, and weight. Body mass index (BMI) was calculated as weight (kg) divided by height squared (m^2^), and the duration of disease (from the time of symptom onset to the time of inclusion in this study) was also recorded. Patients with GHPA were followed prospectively for changes in serum IGF-1, UA, and other biochemical parameters levels at 3, 6, and 12 months postoperatively. Patients with hypopituitarism were treated with appropriate hormone replacement therapy. Adjuvant therapy was administered to patients with inadequate disease control based on the size and location of residual tumors, biochemical parameters, available medical therapy, and the patient’s medical condition and preference. The criteria for remission of GHPA were: fasting serum GH < 2.5 ng/mL or GH < 1 ng/mL on a GH suppression test after administration of a glucose load; and IGF-1 levels decreased to within the normal range that matches age and sex. GHPA patients were divided into a biochemical remission group and a biochemical nonremission group according to their postoperative biochemical remission, and the differences in UA levels between the two groups at 3, 6, and 12 months postoperatively were compared.

### Measurements

Fasting serum GH and IGF-1 levels were measured between 7 am and 9 am by the chemiluminescent immunoassay (IMMULITE 2000 Immunoassay System). The reference range for GH was 3 ng/mL or lower, the upper limit of the measurement range was 40 ng/mL. The IGF-1 level was adjusted according to age and sex and expressed as IGF-1 SDS and the ratio of IGF-1 to the upper limit of normal (ULN) was expressed as IGF-1/ULN. Fasting serum UA, blood urea nitrogen (BUN), creatinine (Cr), estimated glomerular filtration rate (eGFR), alanine transaminase (ALT), aspartate transaminase (AST), total protein (TP), albumin (ALB), fasting blood glucose (FBG), and other biochemical parameters were measured simultaneously according to standard laboratory methods. And the homeostasis model assessment of insulin resistance (HOMA-IR) was calculated as fasting insulin concentration (μU/mL) × FBG (mmol/L)/22.5 (16).

### Statistical analysis

IBM SPSS Statistics for Windows, version 23 (IBM Corp., Armonk, NY, USA), R statistical software, version 4.2.1 (https://www.r-project.org), and GraphPad Prism 8 (GraphPad Software, Inc., La Jolla, CA, USA) was used for statistical analysis and graphing in our study. Normality tests were performed for continuous variables, none of which conformed to a normal distribution, expressed as median (interquartile range), and the Mann-Whitney test was used for comparisons between groups. Spearman’s correlation tests were performed at baseline to examine the correlation between IGF-1 and other anthropometric and biochemical variables, while the partial correlation was used after adjusting for sex, age, and BMI. After adjusting for sex, age, and BMI, univariate and multivariate linear regression analysis was conducted to investigate the independent association between UA and IGF-1 at baseline. Variance inflation factor (VIF) was used to diagnose multicollinearity between the independent variables. Categorical variables were expressed as percentages (%), and the chi-square test was used for comparison between groups. Differences were considered significant if *P* < 0.05.

## Results

### Baseline clinical characteristics of the participants

The baseline clinical characteristics of the enrolled patients are shown in **Table 1**. The total 424 GHPA patients included 47.17% males and 52.83% females. Compared to female GHPA patients, male GHPA patients had lower age and higher UA, BMI, IGF-1, IGF-1 SDS, IGF-1/ULN, BUN, Cr, eGFR, ALT, and ALB.

**Table 1:** Baseline clinical characteristics of the study participants.

Variable	Total (n=424)	Male (n=200)	Female (n=224)	*P* value
Age (year)	40.00 (31.00-49.00)	38.00 (30.25-47.00)	41.50 (33.00-51.00)	0.003^**^
Duration (month)	35.00 (11.25-70.00)	33.50 (11.25-78.50)	36.00 (11.25-60.00)	0.825
BMI (kg/m^2^)	26.06 (23.88-27.64)	26.18 (24.80-28.68)	25.98 (23.24-27.07)	<0.001^***^
DM (%)	84 (19.81)	38 (19.00)	46 (20.54)	0.692
HTN (%)	89 (20.99)	37 (18.50)	52 (23.21)	0.234
GH (ng/mL)	17.25 (8.20-40.00)	17.25 (8.87-40.00)	17.20 (8.02-31.68)	0.087
IGF-1 (ng/mL)	753.00 (608.00-899.00)	753.00 (668.00-992.25)	711.50 (552.50-797.25)	<0.001^***^
IGF-1 SDS	7.26 (4.99-9.71)	8.13 (5.74-9.96)	6.74 (4.80-9.39)	0.003^**^
IGF-1/ULN	3.22 (2.56-3.87)	3.39 (2.84-4.15)	2.98 (2.47-3.59)	<0.001^***^
FBG (mmol/L)	5.47 (5.06-6.45)	5.51 (5.11-6.31)	5.46 (5.02-6.50)	0.707
BUN (mmol/L)	4.40 (3.53-5.40)	4.60 (3.93-5.88)	4.10 (3.40-5.00)	<0.001^***^
Cr (μmol/L)	45.55 (38.25-56.28)	55.35 (45.03-63.00)	41.25 (36.33-46.35)	<0.001^***^
eGFR (ml/min)	135.47 (125.40-144.45)	135.47 (126.54-150.53)	135.40 (124.43-141.81)	0.019^*^
UA (μmol/L)	306.87 (244.80-357.58)	310.70 (278.73-389.58)	288.60 (229.50-306.87)	<0.001^***^
ALT (U/L)	16.60 (12.30-22.00)	18.70 (14.30-24.03)	14.75 (11.28-20.25)	<0.001^***^
AST (U/L)	15.60 (13.03-18.58)	16.00 (13.33-19.00)	15.20 (12.55-18.10)	0.123
TP (g/L)	71.30 (68.10-74.10)	71.05 (67.93-73.98)	71.50 (68.10-74.28)	0.429
ALB (g/L)	44.30 (42.30-46.00)	45.05 (43.00-46.90)	43.70 (41.60-45.25)	<0.001^***^

BMI, body mass index; DM, diabetes mellitus; HTN, hypertension; GH, growth hormone; IGF-1, insulin-like growth factor-1; IGF-1 SDS, insulin-like growth factor-1 standard deviation score; IGF-1/ULN, the ratio of IGF-1 to the upper limit of normal; FBG, fasting blood glucose; BUN, blood urea nitrogen; Cr, creatinine; eGFR, estimated glomerular filtration rate; UA, uric acid; ALT, alanine transaminase; AST, aspartate transaminase; TP, total protein; ALB, albumin; ^***^ *P* values < 0.001; ^**^ *P* values < 0.01; ^*^ *P* values < 0.05.

HUA was defined as serum UA > 420 μmol/L (17). Of the 424 patients included, a total of 40 (9.43%) patients developed HUA, including 34 males, 17.00% (34/200) of male patients, and 6 females, 2.68% (6/224) of female patients. We divided GHPA patients into with HUA and without HUA groups for comparison, as shown in **Table 2**. The results showed that the HUA patients had lower age and higher BMI, IGF-1, BUN, Cr, ALT, TP, and ALB.

**Table 2:** Comparison of baseline clinical characteristics of patients in the GHPA with HUA and without HUA groups.

Variable	Total (n=424)	GHPA with HUA (n=40)	GHPA without HUA (n=384)	*P* value
Sex (M/F)	200/224	34/6	166/218	<0.001^***^
Age (year)	40.00 (31.00-49.00)	33.00 (29.00-43.75)	40.00 (31.25-50.00)	0.017^*^
Duration (month)	35.00 (11.25-70.00)	33.50 (11.25-59.00)	35.00 (11.25-71.00)	0.758
BMI (kg/m^2^)	26.06 (23.88-27.64)	27.11 (26.00-29.89)	26.06 (23.68-27.44)	0.001^**^
DM (%)	84 (19.81)	4 (10.00)	80 (20.83)	0.102
HTN (%)	89 (20.99)	5 (12.50)	84 (21.88)	0.166
GH (ng/mL)	17.25 (8.20-40.00)	15.45 (5.61-40.00)	17.35 (8.27-39.48)	0.910
IGF-1 (ng/mL)	753.00 (608.00-899.00)	838.50 (626.25-1131.75)	753.00 (602.75-882.75)	0.010^*^
IGF-1 SDS	7.26 (4.99-9.71)	7.32 (4.72-9.65)	7.26 (5.00-9.71)	0.879
IGF-1/ULN	3.22 (2.56-3.87)	3.35 (2.58-4.30)	3.22 (2.56-3.83)	0.292
FBG (mmol/L)	5.47 (5.06-6.45)	5.55 (5.02-6.04)	5.47 (5.06-6.48)	0.583
BUN (mmol/L)	4.40 (3.53-5.40)	5.25 (3.90-6.08)	4.35 (3.50-5.30)	0.046^*^
Cr (μmol/L)	45.55 (38.25-56.28)	56.40 (44.53-71.00)	45.00 (37.70-54.98)	<0.001^***^
eGFR (ml/min)	135.47 (125.40-144.45)	135.49 (122.45-149.09)	135.47 (125.40-143.99)	0.686
UA (μmol/L)	306.87 (244.80-357.58)	475.25 (435.88-519.65)	304.00 (239.13-324.08)	<0.001^***^
ALT (U/L)	16.60 (12.30-22.00)	19.05 (14.93-24.18)	16.35 (12.10-21.85)	0.026^*^
AST (U/L)	15.60 (13.03-18.58)	16.60 (13.80-20.00)	15.55 (13.00-18.40)	0.199
TP (g/L)	71.30 (68.10-74.10)	73.00 (70.58-76.73)	71.10 (67.80-73.90)	0.002^**^
ALB (g/L)	44.30 (42.30-46.00)	45.75 (43.90-47.70)	44.20 (42.23-45.88)	0.001^**^

GHPA, growth hormone-secreting pituitary adenoma; HUA, hyperuricemia; BMI, body mass index; DM, diabetes mellitus; HTN, hypertension; GH, growth hormone; IGF-1, insulin-like growth factor-1; IGF-1 SDS, insulin-like growth factor-1 standard deviation score; IGF-1/ULN, the ratio of IGF-1 to the upper limit of normal; FBG, fasting blood glucose; BUN, blood urea nitrogen; Cr, creatinine; eGFR, estimated glomerular filtration rate; UA, uric acid; ALT, alanine transaminase; AST, aspartate transaminase; TP, total protein; ALB, albumin; ^***^ *P* values < 0.001; ^**^ *P* values < 0.01; ^*^ *P* values < 0.05.

### Correlation between IGF-1 and anthropometric and biochemical variables at baseline

We analyzed the relationship between IGF-1 levels and anthropometric and biochemical variables at baseline, as shown in **Table 3**. IGF-1 levels were significantly positively correlated with UA in unadjusted (r = 0.250, *P* < 0.001) and adjusted (r = 0.122, *P* = 0.012) correlation analyses. Other variables, including GH, TP, and ALB, were also significantly positively correlated with IGF-1 levels in the adjusted correlation analysis (*P* < 0.001). Among the 424 patients with GHPA included in this study, a total of 152 patients completed lipid testing. The results of correlation analysis showed that IGF-1 was positively correlated with triglyceride (TG) and negatively correlated with high-density lipoproteins-cholesterol (HDL-c) (r = 0.260, *P* = 0.001; r = -0.259, *P* = 0.001, respectively, data not shown).

**Table 3:** Correlation between IGF-1 and anthropometric and biochemical variables at baseline.

Variable	Unadjusted model		Adjusted model	
	r	*P*	r	*P*
Sex	0.263	<0.001^***^		
BMI (kg/m^2^)	0.194	<0.001^***^		
Age (year)	-0.256	<0.001^***^		
Duration (month)	-0.041	0.399	-0.070	0.154
GH (ng/mL)	0.275	<0.001^***^	0.224	<0.001^***^
FBG (mmol/L)	0.081	0.098	0.080	0.099
BUN (mmol/L)	-0.033	0.502	-0.024	0.620
Cr (μmol/L)	0.114	0.018^*^	-0.028	0.564
eGFR (ml/min)	0.194	<0.001^***^	0.037	0.449
UA (μmol/L)	0.250	<0.001^***^	0.122	0.012^*^
ALT (U/L)	0.143	0.003^**^	0.043	0.384
AST (U/L)	0.059	0.226	-0.009	0.848
TP (g/L)	0.184	<0.001^***^	0.174	<0.001^***^
ALB (g/L)	0.268	<0.001^***^	0.193	<0.001^***^

IGF-1, insulin-like growth factor-1; BMI, body mass index; GH, growth hormone; FBG, fasting blood glucose; BUN, blood urea nitrogen; Cr, creatinine; eGFR, estimated glomerular filtration rate; UA, uric acid; ALT, alanine transaminase; AST, aspartate transaminase; TP, total protein; ALB, albumin; ^***^ *P* values < 0.001; ^**^ *P* values < 0.01; ^*^ *P* values < 0.05.

### Univariate and multivariate linear regression analysis of UA with anthropometric and biochemical variables at baseline

Univariate linear regression was used to analyze the relationship between UA and anthropometric and biochemical variables at baseline, and the results are shown in **Table 4**. After adjusting for sex, age, and BMI, combined with DM, IGF-1, BUN, Cr, eGFR, and TP were influential factors of UA.

**Table 4:** Univariate linear regression analysis of the relationship between UA and anthropometric and biochemical variables.

Variable	Unadjusted model			Adjusted model		
	β	SE	*P*	β	SE	*P*
Sex	57.492	8.107	<0.001^***^			
Age (year)	-1.430	0.345	<0.001^***^			
BMI (kg/m^2^)	5.613	1.191	<0.001^***^			
DM	33.702	10.615	0.002^**^	25.041	10.146	0.014^*^
HTN	-7.761	10.506	0.460	10.448	11.000	0.343
Duration (month)	-0.058	0.074	0.428	-0.047	0.071	0.508
GH (ng/mL)	0.019	0.305	0.949	-0.372	0.296	0.210
IGF-1 (ng/mL)	0.094	0.018	<0.001^***^	0.046	0.018	0.012^*^
IGF-1 SDS	1.559	1.243	0.210	2.517	1.339	0.061
IGF-1/ULN	12.657	4.178	0.003^**^	6.650	4.032	0.100
FBG (mmol/L)	-2.282	1.458	0.118	-2.329	1.350	0.085
BUN (mmol/L)	8.058	3.084	0.009^**^	9.614	3.195	0.003^**^
Cr (μmol/L)	2.557	0.294	<0.001^***^	2.270	0.327	<0.001^***^
eGFR (ml/min)	0.030	0.218	0.889	-1.376	0.283	<0.001^***^
ALT (U/L)	0.692	0.298	0.021^*^	0.416	0.278	0.135
AST (U/L)	0.516	0.466	0.269	0.408	0.431	0.343
TP (g/L)	2.683	0.792	<0.001^***^	2.416	0.738	0.001^**^
ALB (g/L)	5.323	1.331	<0.001^***^	2.308	1.317	0.080

UA, uric acid; BMI, body mass index; DM, diabetes mellitus; HTN, hypertension; GH, growth hormone; IGF-1, insulin-like growth factor-1; IGF-1 SDS, insulin-like growth factor-1 standard deviation score; IGF-1/ULN, the ratio of IGF-1 to the upper limit of normal; FBG, fasting blood glucose; BUN, blood urea nitrogen; Cr, creatinine; eGFR, estimated glomerular filtration rate; ALT, alanine transaminase; AST, aspartate transaminase; TP, total protein; ALB, albumin; ^***^ *P* values < 0.001; ^**^ *P* values < 0.01; ^*^ *P* values < 0.05.

Multivariate linear regression analysis was performed with UA as the dependent variable. When Cr and eGFR were simultaneously included as independent variables in the equation, the calculated VIF > 10, and the presence of multicollinearity was considered, so we listed the four models separately, and the results are shown in **Table 5**. In model 1, IGF-1, BUN, Cr, TP, and combined with DM were included as independent variables, and the results showed that IGF-1, Cr, TP, and combined with DM were all influential factors of UA. In model 2, after including sex, age, and BMI as confounders in the equation, the results showed that IGF-1, Cr, and TP remained independent influences on UA levels. Whereas in model 3, IGF-1, BUN, eGFR, TP, and combined with DM were included as independent variables, and the results showed that IGF-1, BUN, TP, and combined with DM were the influencing factors of UA levels. In model 4, after including sex, age, and BMI as confounders, the results showed that IGF-1, BUN, eGFR, TP, and combined with DM were independent influences on UA levels. In all of the above models, IGF-1 was an independent influence on UA levels. As the level of IGF-1 increased, the level of UA gradually increased.

**Table 5:** Multivariate linear regression analysis of the relationship between UA and anthropometric and biochemical variables.

	Model 1			Model 2			Model 3			Model 4		
	β	SE	*P*	β	SE	*P*	β	SE	*P*	β	SE	*P*
IGF-1	0.072	0.017	<0.001^***^	0.041	0.017	0.018^*^	0.087	0.018	<0.001^***^	0.041	0.018	0.020^*^
BUN	0.397	3.082	0.898	4.047	3.164	0.201	10.626	3.283	0.001^**^	6.331	3.214	0.049^*^
Cr	2.445	0.315	<0.001^***^	2.123	0.342	<0.001^***^	–	–	–	–	–	–
eGFR	–	–	–	–	–	–	-0.014	0.230	0.952	-1.208	0.290	<0.001^***^
TP	2.382	0.726	0.001^**^	2.211	0.707	0.002^**^	1.824	0.775	0.019^*^	2.119	0.724	0.004^**^
DM	20.998	9.814	0.033^*^	14.604	9.726	0.134	35.804	10.296	<0.001^***^	20.354	9.879	0.040^*^

Model 1: IGF-1, BUN, Cr, TP, and combined with DM as independent variables, adjusted no variables.

Model 2: Model 1, adjusted for age, sex, and BMI.

Model 3: IGF-1, BUN, eGFR, TP, and combined with DM as independent variables, adjusted no variables.

Model 4: Model 3, adjusted for age, sex, and BMI.

UA, uric acid; IGF-1, insulin-like growth factor-1; BUN, blood urea nitrogen; Cr, creatinine; eGFR, estimated glomerular filtration rate; TP, total protein; DM, diabetes mellitus; BMI, body mass index; ^***^ *P* values < 0.001; ^**^ *P* values < 0.01; ^*^ *P* values < 0.05.

-, the parameter is not included in the equation.

Besides, fasting insulin levels were tested in only 69 of the 424 patients with GHPA included at baseline, and no significant correlation was observed between UA and fasting insulin or HOMA-IR levels (r = 0.193, *P* = 0.111, and r = 0.037, *P* = 0.761, respectively, data not shown).

### Postoperative changes in IGF-1, UA, and other biochemical parameters levels

**Figure 1:**
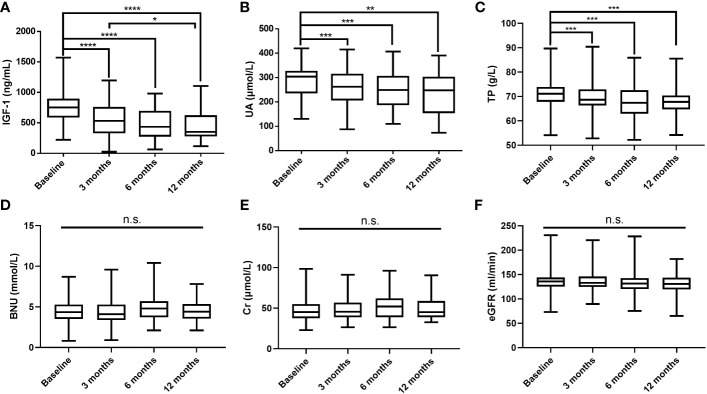
Postoperative changes in IGF-1 **(A)**, UA **(B)**, TP **(C)**, BUN **(D)**, Cr **(E)**, and eGFR **(F)** levels. IGF-1, insulin-like growth factor-1; UA, uric acid; TP, total protein; BUN, blood urea nitrogen; Cr, creatinine; eGFR, estimated glomerular filtration rate; ****P values < 0.0001; ***P values < 0.001; **P values < 0.01; *P values < 0.05; n.s., not significant.

After excluding GHPA patients with HUA and receiving urate-lowering treatment, the changes in IGF-1, UA, and other biochemical parameters levels were measured at 3 months (n = 126), 6 months (n = 51), and 12 months (n = 34) after surgery, and the results are shown in **Figure 1**. As IGF-1 levels decreased, UA and TP levels gradually decreased, while BUN, Cr, and eGFR levels did not change significantly.

**Figure 2:**
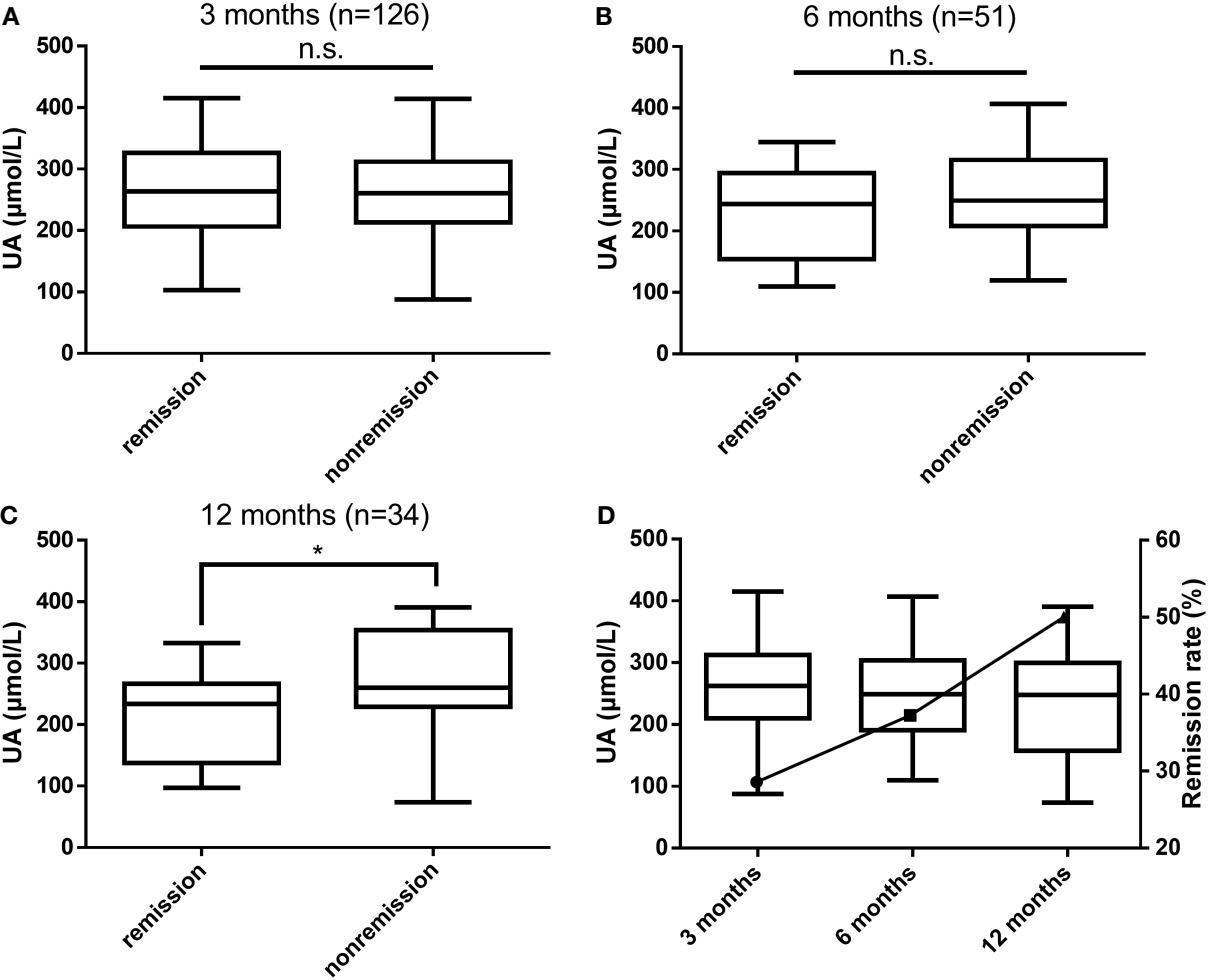
Differences in UA levels between the biochemical remission and nonremission groups at 3 months **(A)**, 6 months **(B)**, and 12 months **(C)** postoperatively and the relationship between biochemical remission rates and UA levels **(D)**. UA, uric acid; n.s., not significant; *P values < 0.05.

According to postoperative biochemical remission, the patients were divided into the biochemical remission group and the nonremission group, and patients who did not achieve biochemical remission after surgery were treated with drugs, a second surgery, or radiation therapy. The differences in UA levels between the two groups at 3, 6, and 12 months after surgery were compared, as shown in **Figures 2A–C**. At 3 months postoperatively, the biochemical remission rate was 28.57% (36/126), and no significant difference in UA levels between the two groups was observed. At 6 months postoperatively, the remission rate was 37.25% (19/51), and a trend of lower UA levels in the biochemical remission group than in the nonremission group could be observed. At 12 months postoperatively, the remission rate was 50.00% (17/34), and the UA levels in the biochemical remission group were significantly lower than those in the nonremission group (*P* = 0.038). With the rate of biochemical remission increased, UA levels gradually decreased (**Figure 2D**).

## Discussion

Circulating high levels of GH/IGF-1 in patients with GHPA can affect all aspects of body metabolism, including the synthesis and breakdown of proteins, fats, and carbohydrates, as well as the metabolism of sodium, calcium, and phosphorus in the kidneys (3). Previous literature reported the prevalence of DM in GHPA patients in the range of 12%-37.6% (18–20) and hypertension (HTN) in 29.4% (21–24), which was close to the prevalence of DM and HTN in the 424 patients with GHPA included in this study. In terms of lipid metabolism, previous studies have shown that patients with active GHPA have increased TG and decreased HDL-c (25), which was consistent with the results of the data analysis in this study. These metabolic-related complications including DM, HTN, and abnormal lipid metabolism lead to a significantly higher rate of cardiovascular disease mortality in patients with GHPA, which remains 1.32 times higher than that of the general population even in patients undergoing transsphenoidal surgery (3). Serum UA is the end-product of purine metabolism via xanthine oxidoreductase in the body and is excreted primarily by the kidney and intestinal tract. Elevated UA is associated with a variety of adverse health outcomes, including HTN, obesity, dyslipidemia, atherosclerosis, DM, chronic kidney disease, stroke, and cardiovascular disease (CVD) (26). Several large population-based studies have shown that HUA is associated with an increased risk of cardiovascular events and mortality (27–29). Thus, there is an urgent need for early screening and preventative strategies for HUA. The 2018 European Society of Cardiology (ESC) and European Society of Hypertension (ESH) Guidelines (30) and the 2020 International Society of Hypertension (ISH) Guidelines (31) on Hypertension have included serum UA as an additional risk factor for CVD, demonstrating the importance of detecting UA in the presence of a risk of a cardiovascular event, and therefore it is necessary to explore changes in UA levels in patients with GHPA.

However, fewer studies reported the effect of elevated GH/IGF-1 levels on UA metabolism in GHPA patients. Our findings identified an independent correlation between IGF-1 and UA levels in patients with GHPA, which was confirmed by multiple regression analysis after adjusting for age, sex, and BMI. More importantly, at 12 months postoperatively, serum UA levels decreased significantly with decreasing IGF-1, and UA levels were significantly lower in the biochemical remission group than in the nonremission group. This indicates that UA levels in patients with GHPA are related to disease activity, and UA levels are significantly higher in patients with active GHPA and decrease significantly after treatment along with disease control. Our findings also showed that men with GHPA had significantly higher UA levels than women and that the majority of GHPA patients who developed HUA were men (85%). Compared with GHPA patients without HUA, patients with combined HUA had lower age and higher BMI. Therefore, we believe that in young, obese male GHPA patients, more attention needs to be paid to monitoring UA levels and being alert to the development of HUA. For those patients with GHPA who develop HUA, clinicians should provide health education and recommend lifestyle changes, including limiting high-purine and fructose-containing foods and using urate-lowering treatment as appropriate for the patient’s condition (17, 32, 33). Meanwhile, it is necessary to take active measures to control GHPA, reduce IGF-1 levels to achieve biochemical remission, and dynamically follow up changes in UA levels to improve the patient’s prognosis.

Current research is controversial regarding the relationship between GH/IGF-1 and UA levels. In a study of 1430 adult non-diabetic subjects, results showed that low circulating IGF-1 levels were associated with high serum UA (9). A study revealed a non-linear relationship between UA concentrations and IGF-1 SDS in children and adolescents with ISS, where IGF-1 SDS was positively correlated with appropriate serum UA concentrations, while too high or too low serum UA levels were associated with lower IGF-1 SDS values (11). Another longitudinal study of 182 prepubertal ISS children treated with rhGH for 30 months showed that UA was significantly associated with height SDS after controlling for sex, age, and BMI SDS, and no statistical correlation was observed despite similar trends in increasing IGF-1 SDS and UA levels (34). In boys with obesity and NAFLD, no change was observed in UA levels at months 3 and 6 of rhGH administration (12). But our study showed that UA was positively correlated with circulating high levels of IGF-1 in patients with GHPA. The decrease in IGF-1 levels was accompanied by a significant decrease in UA levels as the disease was controlled, but further *in vitro* studies are needed to confirm our findings.

The mechanism by which IGF-1 affects UA levels remains unclear. JAK2-STAT5 signaling is the classical pathway involved in regulating IGF-1 gene expression (35). An *in vitro* study incubated human hepatoma cells with UA for 24 or 48 h in the presence of GH and observed a 21% and 26% reduction, respectively, in GH-stimulated IGF-1 mRNA expression (*P* = 0.020 and *P* = 0.012, respectively), suggesting that exposure of human hepatoma cells to UA impairs the ability of GH to stimulate JAK2-STAT5 signaling (9). Another study showed that IGF-1 reduces UA by activating urate secretory transporters and inhibiting insulin’s action (36). However, a retrospective longitudinal cohort study that collected blood samples from 1506 Japanese individuals and analyzed the data to determine the association between IGF-1 genotype and biochemical parameters of patients showed no association between IGF-1 genotype and serum UA levels (37).

Serum UA levels and insulin resistance are significantly correlated and the reciprocal causation. Recent studies have found that UA directly induces insulin resistance and insulin signaling impairment *in vitro* and *in vivo* (38–40). A study showed that patients with HUA are at significantly higher risk of developing insulin resistance, and that elevated UA leading to insulin resistance may be related to the ability of high UA to increase insulin secretion in all temporal phases of pancreatic β-cells (41). Prolonged hyperinsulinemia leads to elevated circulating UA levels through increased anabolism of UA and activation of proximal tubular urate reabsorption (42, 43). GH is an insulin antagonist hormone that causes insulin resistance in adipose and skeletal muscle. IGF-1 activates insulin/IGF-1 heterotrimeric receptors in adipose, muscle, and liver to exert insulin-like effects. Thus GH/IGF-1 has insulin-like and antagonistic insulin effects and can regulate tissue sensitivity to insulin (44). Studies have demonstrated that GH/IGF-1 levels in GHPA patients are closely associated with insulin resistance (45, 46). Therefore, we consider that in GHPA patients, high levels of GH/IGF-1 lead to elevated circulating UA levels through the induction of insulin resistance. However, no significant correlation between UA and fasting insulin levels or HOMA-IR levels was observed as a result of data analysis in this study. We considered that HOMA-IR may not be able to accurately assess the severity of insulin resistance in the high GH/IGF-1 state, and subsequent expansion of sample size and hyperinsulinemic-euglycemic clamp tests and *in vitro* studies are needed to further investigate the relationship between GH/IGF-1 and UA levels and insulin resistance in GHPA patients.

This study has certain limitations. Firstly, UA levels may be influenced by the purine and fructose content of the diet, alcohol intake, and genetic factors, but due to the limitations of the retrospective study, it is difficult for us to accurately collect the purine and fructose content of the patients’ diets and their alcohol intake. Besides, some components of the metabolic syndrome, such as lipids, were not well collected. Moreover, in this study, we did not compare the changes in UA levels in patients with GHPA to a control group matched for age, sex, kidney, and metabolic variables, and a further control group will be established to validate our findings. Finally, significantly lower UA levels were observed in the biochemical remission group compared to the biochemical nonremission group at 12 months postoperatively. However, no significant difference was observed at 3 and 6 months postoperatively, probably due to the short follow-up period, the low proportion of patients achieving biochemical remission, and the small sample size. Further increase in the number of follow-up patients is needed to confirm our conclusion.

## Conclusions

It is the first study that investigates the relationship between IGF-1 levels and UA in patients with GHPA. High levels of IGF-1 lead to elevated UA levels, and after treatment, UA gradually decreases as IGF-1 levels decrease, indicating that UA levels are correlated with disease activity in patients with GHPA. Therefore, during the comprehensive treatment of GHPA, besides paying attention to metabolic complications such as blood glucose, blood pressure, and lipids, changes in UA levels should also be taken into account. When HUA develops, clinicians should instruct patients to change their lifestyles and administer urate-lowering treatment as appropriate according to their conditions, while taking active measures to control GHPA and lowering IGF-1 levels to achieve biochemical remission, and dynamically monitoring the changes in UA levels to improve patients’ prognosis.

## Data availability statement

The raw data supporting the conclusions of this article will be made available by the authors, without undue reservation.

## Ethics statement

The studies involving humans were approved by the Human Research Ethics Committee of Beijing Tiantan Hospital, Capital Medical University (No. KY2022-024-01). The studies were conducted in accordance with the local legislation and institutional requirements. Written informed consent for participation was provided from the participants or the participants’ legal guardians/next of kin in accordance with the national legislation and institutional requirements.

## Author contributions

CM collected data, conducted statistical analysis, and wrote the first draft, HC provided statistical guidance, FW, YG, and YW provided writing guidance, TT helped collect data, and LZ guided the research direction and revised the article. All authors contributed to the article and approved the submitted version.
